# Automated characterization of flowering dynamics in rice using field-acquired time-series RGB images

**DOI:** 10.1186/s13007-015-0047-9

**Published:** 2015-02-13

**Authors:** Wei Guo, Tokihiro Fukatsu, Seishi Ninomiya

**Affiliations:** Institute for Sustainable Agro-ecosystem Services, Graduate School of Agricultural and Life Sciences, The University of Tokyo, 1-1-1. Midori-cho, Nishi-Tokyo, Tokyo 188-0002 Japan; National Agriculture and Food Research Organization, 3-1-1 Kannondai, Tsukuba, Ibaraki 305-8666 Japan

**Keywords:** Time-series RGB image, SIFT, BoVWs, SVM

## Abstract

**Background:**

Flowering (spikelet anthesis) is one of the most important phenotypic characteristics of paddy rice, and researchers expend efforts to observe flowering timing. Observing flowering is very time-consuming and labor-intensive, because it is still visually performed by humans. An image-based method that automatically detects the flowering of paddy rice is highly desirable. However, varying illumination, diversity of appearance of the flowering parts of the panicles, shape deformation, partial occlusion, and complex background make the development of such a method challenging.

**Results:**

We developed a method for detecting flowering panicles of rice in RGB images using scale-invariant feature transform descriptors, bag of visual words, and a machine learning method, support vector machine. Applying the method to time-series images, we estimated the number of flowering panicles and the diurnal peak of flowering on each day. The method accurately detected the flowering parts of panicles during the flowering period and quantified the daily and diurnal flowering pattern.

**Conclusions:**

A powerful method for automatically detecting flowering panicles of paddy rice in time-series RGB images taken under natural field conditions is described. The method can automatically count flowering panicles. In application to time-series images, the proposed method can well quantify the daily amount and the diurnal changes of flowering during the flowering period and identify daily peaks of flowering.

**Electronic supplementary material:**

The online version of this article (doi:10.1186/s13007-015-0047-9) contains supplementary material, which is available to authorized users.

## Background

The dynamics of flowering is an important trait for paddy rice and affects the maturation timing of rice grain [1,2]. Great effort is invested in observing flowering time. Diurnal variance in flowering time is also important because heat reduces pollen fertility and pollination efficiency, reducing yield and degrading grain quality. Facing global warming, rice breeders are now trying to find early-morning flowering lines to avoid heat at the time of flowering [3,4]. The search for early-morning-flowering lines requires observers to remain in fields, for several hours daily, starting early morning.

Machine learning and digital image processing techniques are becoming readily available for field-based agronomic applications. For example, methods for measuring or estimating crop growth parameters such as canopy coverage, leaf area index, and plant height [5-12] and for monitoring crop growth status [13-15] have been recently proposed. In particular, methods for extracting the phenotypic characteristics of specific plant organs (leaf, fruit, flower, grain, etc.) have been helpful for researchers and breeders attempting to understand the performance of crop genetic resources [16-20]. In view of such innovative applications of image analysis for crops, an image-based method that automatically detects and quantifies the flowering behavior of paddy rice appears feasible.

**Figure 1:**
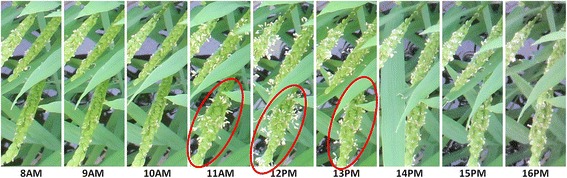
**An example of the same panicles**’ **appearance in one day.** The daily active flowering time is short. In this example, active flowering starts around 11:00 and lasts until anthers begin shrinking around 13:00. The red elliptic circles indicate examples of actively flowering panicles.

**Figure 2:**
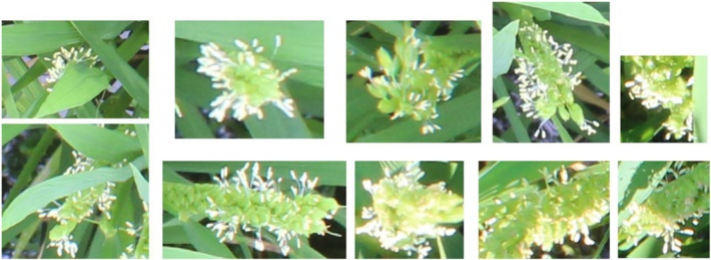
**Various appearances of flowering panicles.**

**Figure 3:**
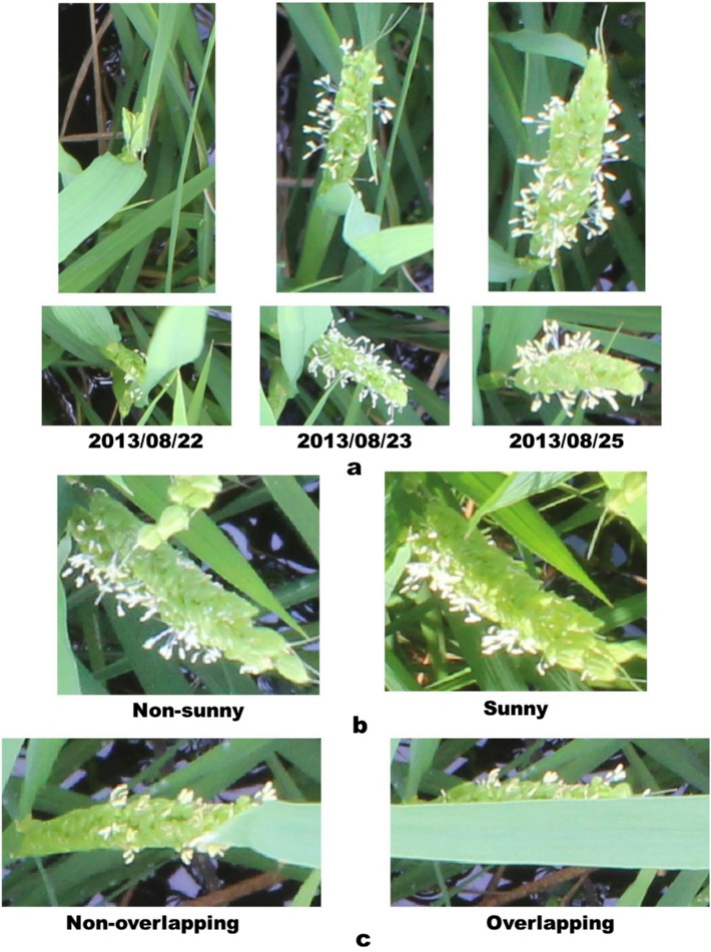
**Changes in the appearance of identical flowering panicles.** **(a)** Images of two identical flowering panicles taken over three consecutive days. Physical size and shape change owing to growth; **(b)** Images of an identical flowering panicle. The appearance changes under different light conditions; **(c)** Images of an identical flowering panicle. The appearance is changed by an overlapping leaf.

Generally, flowering in paddy rice occurs by anther extrusion between the opening and closing of the spikelet. Active flowering generally lasts for 1–2.5 h daily during the reproductive phase, and it is very sensitive to external environmental factors such as temperature, solar radiation, etc. [21,22]. For example in Figure 1 active flowering is observed only in the image acquired at around 12 PM. Moreover, because the crop grows under natural conditions, varying illumination, diverse orientations, various appearances of panicles, shape deformation by wind and rain, partial occlusion, and complex background make image-based methods challenging. Figure 2 shows examples of various appearances of flowering panicles of rice, and Figure 3 demonstrates how they change with growth and the external environment. Figure 3a shows physical size and shape changes due to growth in two panicles taken over three days. Figure 3b and c show images taken within a 5-min interval may be very different because of color changes under natural light conditions and shape changes due to leaf overlapping.

In this study, we combined a local feature descriptor, the scale-invariant feature transform (SIFT) [23], an image representation method, the bag of visual words (BoVWs) [24,25], and a machine learning model, the support vector machine (SVM) [26] to overcome these difficulties, and attempted to develop a model able to detect flowering panicles of paddy rice in normal RGB images taken under natural field conditions. The method is based on generic object-recognition technology, which is still challenging in machine vision. We evaluated the performance of the proposed method by monitoring the diurnal/daily flowering pattern and the flowering extent of paddy rice during the flowering period. Although some methods such as the color based method for lesquerella [27] and the spectral reflectance based method for winter wheat [28] have been studied to identify flowers under natural condition, no digital image-based identification method of paddy rice flowering has been proposed to date.

## Results

We acquired two independent time series images of two paddy rice varieties, *Kinmaze* and *Kamenoo* and provided three datasets, Dataset 1, Dataset 2 and Dataset 3 to verify the flowering identification capabilities of the proposed method. The images were taken every 5 minutes from 8:00 to 16:00 between days 84 and 91 after transplanting considering the flowering period of the varieties. Dataset 1 and Dataset 3 are composed of the original 645 and 768 full size images of *Kinmaze* and *Kamenoo* respectively whereas Dataset 2 is composed of the central parts of the images cropped from Dataset 1. A total of 700 image patches sampled from 21 images of Dataset 1 were used to train the support vector machine (SVM) model for detecting the flowering in the proposed method. The 21 images were removed from Dataset 1 and Dataset 2 when the datasets were used for the model verifications.

**Figure 4:**
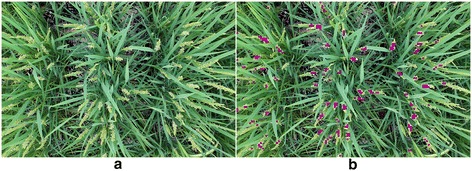
**An example of flowering panicle detection of Dataset 1** **(** **variety** **,** ***Kinmaze*** **)** **by the method developed in this study.** **(a)** Original image from Dataset 1; **(b)** Each violet block indicates a sliding window in which part of a flowering panicle was detected.

**Figure 5:**
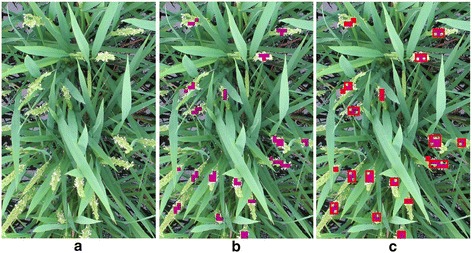
**An example of flowering panicle detection of Dataset 2 by the method developed in this study.** **(a)** Original image from Dataset 2; **(b)** Each violet block indicates a sliding window in which part of a flowering panicle was detected. **(c)** Each red-outlined rectangle indicates a region of connected blocks.

**Figure 6:**
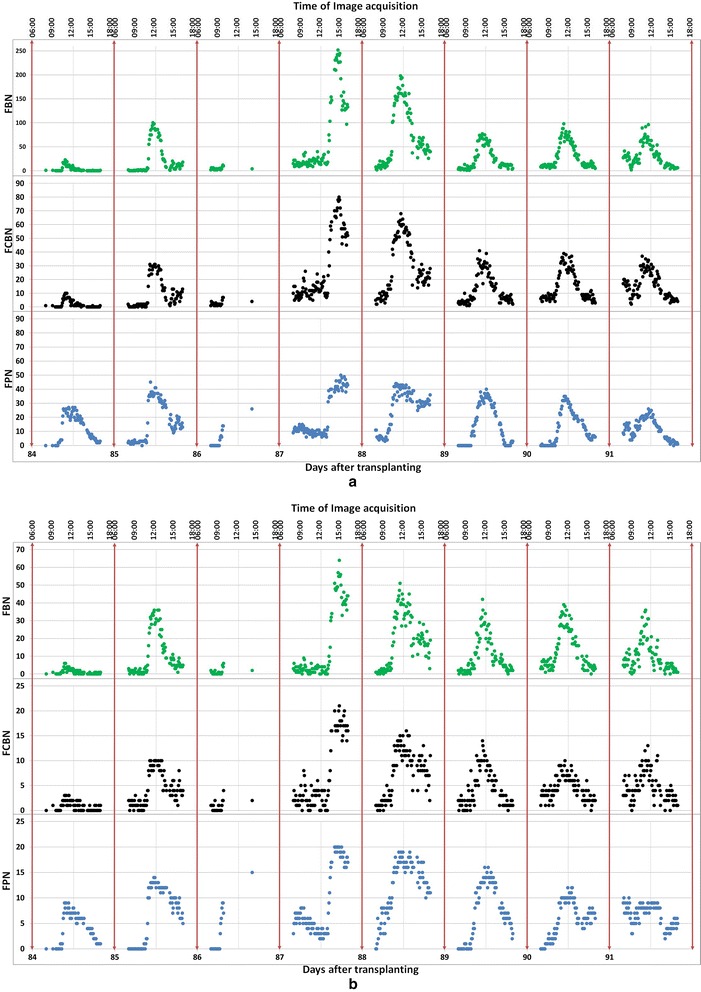
**Comparison of manually and automatically determined numbers of flowering panicles of Dataset 1 and Dataset 2.** FBN: the number of the blocks which are judged to contain the flowering parts of panicles; FCBN: the number of the regions of connected blocks; FPN: the number of visually counted flowering panicles. **(a)** Dataset 1 for the original full size time series images of *Kinmaze*; **(b)** Dataset 2 for the cropped time series images of *Kinmaze*; The images were acquired every 5 minutes from 08:00 to 16:00 during the flowering period between days 84 and 91 after transplanting. Note that the system sometimes failed to acquire the images, which is particularly obvious on day 86. The failure was caused mainly by unstable network status in the field.

Figures 4 and 5 show examples of the flowering detections in Dataset 1 and Dataset 2. Each small block of violet red color shown in Figures 4b and 5b indicates a sliding window that was assessed as a flowering part (s). The red rectangles in Figure 5c show the regions which surround the connected violet red blocks in Figure 5b and they successfully detected most of the flowering panicles. In additional, a video was provided to demonstrate the detected result during whole experimental period (Additional file 1), the image Datasets and demo matlab Pcode used in this experiment also available on our website^a^. Figure 6a and b show the results of flowering detection between days 84 and 91 after transplanting of Dataset 1 and Dataset 2. Because of transmission errors of the image acquisition system for *Kinmaze*, some of the images, particularly on day 86, are missing. Green, black, and blue circles indicate the number of blocks assigned as flowering parts of panicles (FBN), the number of regions of connected blocks (FCBN), and the number of visually counted flowering panicles (FPN), respectively. The daily flowering patterns shown by FBN and FCBN were similar to the actual number of flowering panicles (FPN). Thus, the method quantified well the daily amount and the diurnal changes of flowering, including identifying the daily peak of flowering. The correlation coefficients between FPN and FBN and between FPN and FCBN were 0.80 and 0.82 respectively for Dataset_1 whereas those for Dataset 2 were 0.81 and 0.82. FCBN was close to FPN, suggesting that FCBN can be used to estimate the number of flowering panicles. Dataset 2 (cropped images) was used to evaluate the influence of the marginal image distortion by the 24 mm wide lens on the detection accuracy but the results did not indicate any influence on the accuracy. Moreover, the curves for FCBN and FBN for Dataset 1 were much smoother than those for Dataset 2, indicating that the larger images could provide more stable detections because of the larger number of the target crops to be detected in an image.

**Figure 7:**
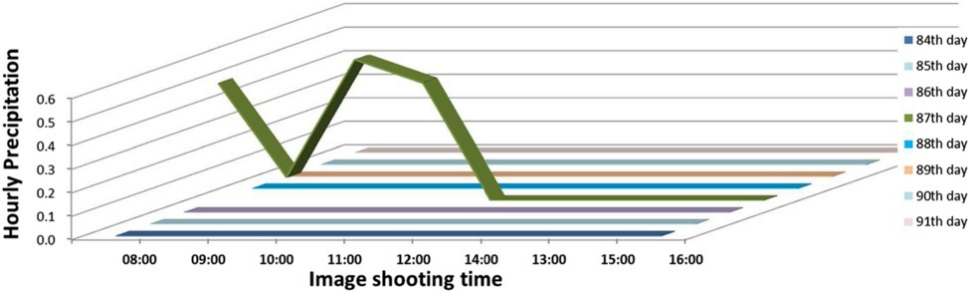
**Hourly precipitation during seven consecutive flowering days from days 84 to 91 after transplanting.** Each line indicates the hourly precipitation from 8:00 to 16:00. Note that it was raining on the morning of day 87 (green line).

Figure 6 shows that the flowering number normally reached a maximum around 12:00 on all days except day 87, when it reached a maximum around 15:00, Rice does not start flowering under rainy conditions [21,29,30] and it was in fact raining on the morning of day 87 (Figure 7). We observed that the rain delayed flowering on this day. This result shows that the proposed method can accurately detect such sensitive physiological responses of rice by identifying flowering timing and extent.

**Figure 8:**
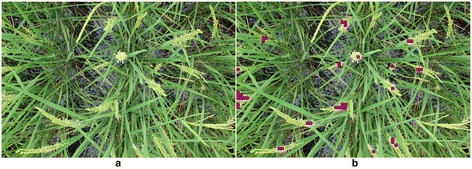
**An example of flowering panicle detection of Dataset 3** **(variety**, ***Kamenoo*** **)** **by the method developed in this study.** **(a)** Original image from Dataset 3; **(b)** Each violet block indicates a window in which part of a flowering panicle was detected.

**Figure 9:**
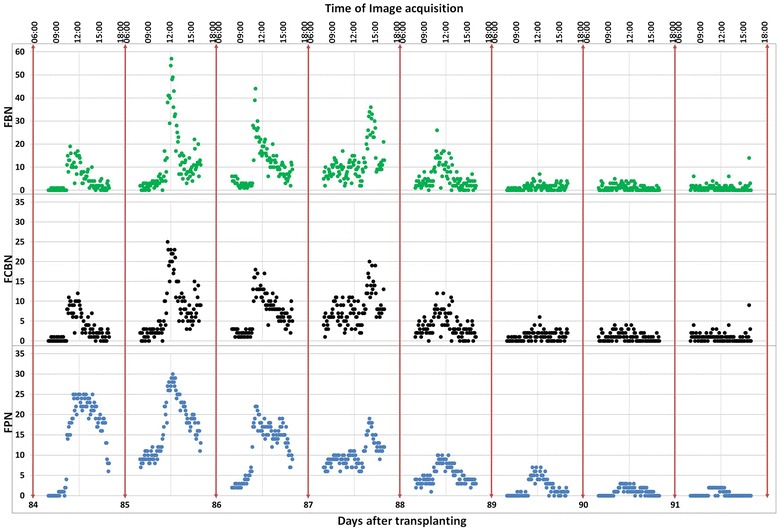
**Comparison of manually and automatically determined numbers of flowering panicles of Dataset 3.** FBN: the number of the blocks which are judged to contain the flowering parts of panicles; FCBN: the number of the regions of connected blocks; FPN: the number of visually counted flowering panicles. The images were acquired every 5 minutes from 08:00 to 16:00 during the flowering period between days 84 and 91 after transplanting.

Dataset 3 (*Kamenoo*) was used to verify the applicability of the above model used for Dataset 1 and Dataset 2. Figures 8 and 9 show the results of the flowering detection on Dataset 3. The correlation coefficients between FPN and FBN and between FPN and FCBN were 0.64 and 0.66, respectively. Although the correlation coefficients were lower than those for Dataset 1 and Dataset 2, the detected patterns of daily and diurnal flowering of *Kamenoo* were well quantified by the model which was trained only by the images of a different variety, *Kinmaze*. Note that the sliding window size used for Dataset 3 to detect the flowering blocks was different from that used for Dataset 1 and Dataset 2 as mentioned in the Method section. We will discuss this point in the Discussion section.

**Figure 10:**
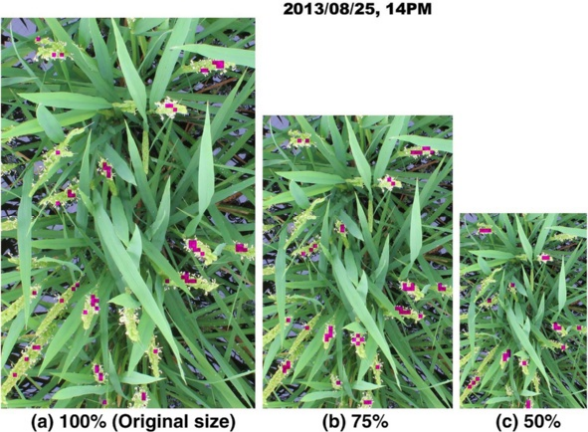
**An example of flowering detection at three different image resolutions.** The resolution of the original image (2001 × 1301 pixels) was reduced by 75% (1501 × 976) and 50% (1001 × 651) and the efficiencies of detection were compared. The detection in the 75% reduction case **(b)** was almost the same as that in the original resolution **(a)** and the correlation coefficient between FPN and FCBN is 0.83, whereas the missed detection in the 50% case **(c)** was obvious and the correlation was 0.73.

Using our computer system (Microsoft Windows 8 PC with a 4-core i7 CPU and 16 GB of memory), the learning process with 600 training image patches (300 flowering and 300 non-flowering) takes approximately 30s. Using only 60 training image patches (30 flowering and 30 non-flowering) takes only 10s. The detection process requires approximately 480 s for each test image of Dataset 1 and Dataset 3 (5184 × 3456 pixels), and 70s for Dataset 2 (2001 × 1301 pixels). Although parallel computing helps us to process four images simultaneously, detection is still computationally expensive (22 ~ 30 h for Dataset 1 and Dataset 3, and 5 ~ 6 h for Dataset 2). We accordingly conducted a preliminary test on Dataset 2 to evaluate the effect of image resolution on the accuracy of the detection, aiming to reduce the computational cost of the method. The original images were resized to 75% and 50% of their original resolution and the accuracy of detection was evaluated (Figure 10). The 75% reduction did not affect accuracy (the correlation coefficient between FPN and FCBN was 0.83), whereas the 50% reduction clearly decreased accuracy (the correlation coefficient was 0.72). These results show that reduction of the test image resolution in an appropriate range reduced computing cost without loss of detection accuracy.

## Discussion

The developed method accurately detected flowering rice panicles in time series of RGB images taken under natural field conditions. It was suggested to use the larger images to cover the larger number of crops, because the detections seemed to be more stable with more crops in a scene. The fact that the distortion of the images in the marginal parts did not influence the accuracy of the detections supported the suggestion. Although, the time series images in this study were acquired regardless of light condition which varied from time to time, the results indicated that the proposed method was rather robust in detecting daily and diurnal flowering patterns. However, we also observed that the detection sometimes failed by specular reflection over panicles caused by extremely strong sunny illumination, degrading the accuracy of the detection. At this moment, we do not have any solution for the issue but it might be a good idea to automatically remove such images with specular reflections as outliers from frequently acquired images. To do so, we need to develop a new algorithm to identify such specular reflections in images.

The general versatility is required for the method to be widely used. As the first step, we examined the applicability of the model trained by the images of *Kinmaze* to a different variety *Kamenoo*. The result indicated that the model could quantify the daily and diurnal patterns of the flowering of the different variety but the correlation coefficients between FPN and FBN and between FPN and FCBN were worse than those for *Kinmaze*. We expect that many factors can cause such degradation. One possible cause of the degradation is the difference in the resolution of the panicle images between two varieties, because the proposed method detects the flowering depending on the spatial features of the images and the spatial features vary with image resolution. Actually, the observed plant heights of *Kinmaze* and *Kamenoo* at the flowering stage were around 107 cm and 145 cm respectively, so that the positions of the panicles of *Kamenoo* were much closer to the camera lens, making the resolution of the panicle images of *Kamenoo* higher. We tried to compensate this issue by adjusting the optimal size of the sliding window to detect the flowering for each variety in a preliminary test. Currently, the adjustment was done *ad hoc* through trial and error and we first need to develop an algorithm to conduct automatic adjustments of the sliding window size. In order to improve the proposed method for its general applicability in paddy rice, we also need to identify other causes of the degradation by using a wide range of varieties.

Generic object recognition is still an important target of pattern recognition studies and continues to be developed. For example, BoVWs count only the occurrences of visual words based on local image features, and ignores location and color information of each feature that may improve the accuracy of the model. For this reason, studies are now focusing on increasing the dimensions of BoVWs by adding more statistical variables such as a vector of locally aggregated descriptors [31], super vector coding [32], a Fisher vector [33], and a vector of locally aggregated tensors [34]. These new concepts have been proposed to accurately recognize and classify large scale images in the real world. We expect that such concepts will contribute to the improvement of our flowering detection method as well as the development of other agricultural applications for high-throughput phenotyping in future studies. Our next step is to improve the accuracy and general versatility of the flowering detection method. To reach this goal, we will also need to identify the optimal quantity and quality of the training image patches in addition to improving the model.

In this study, a camera was fixed, targeting a single plot. However, providing a camera for each plot is impractical when a number of plots are to be observed. Therefore, we are now developing a movable camera system, which can cover several plots only with a single camera. We also expect to use an unmanned aerial vehicle (UAV) to cover a large numbers of plots.

Though we need further improvements of the method as discussed above, the overall results in this study showed a high performance in detecting the flowering panicles of rice. We expect that our method will contribute to practical rice farming management as well as to rice research. Although flowering timing is one of the most important indicators in optimal management and characterization of rice, it is still judged visually, requiring much time. In particular, when a large number of small plots with different flowering timings are to be observed, our method can be especially useful. A typical example is rice breeding, where a large number of plots must be observed efficiently. We expect that the combination of a movable camera system/UAV and the improved version of the proposed method applicable to paddy rice in general will dramatically ease and accelerate the breeding process.

Notably, the diurnal flowering timing of rice is becoming important because of the trend of global warming. The pollination of rice occurs at the timing of spikelet anthesis and the fertility depends strongly on the air temperature at pollination. Therefore, rice varieties flowering early morning before the temperature rises are being sought [3]. In breeding for such varieties, breeders at present must observe many plots of candidate lines continuously for a few hours early morning every day during the expected flowering period. The proposed method, which can accurately detect diurnal flowering timing, is expected to be highly helpful in such cases.

## Methods

### Experimental materials and growth conditions

In this study, the japonica rice (*Oryza sativa* L.) varieties, *Kinmaze* and *Kamenoo*, were used. Seeds were sown on April 26 and transplanted on May 31, 2013 in the field at the Institute for Sustainable Agro-ecosystem Services, University of Tokyo (35°44′22″N, 139°32′34″E and 67 m above the sea level). The area of the experimental field was approximately 250 m^2^, and the planting density was 28 plants/m^2^. From June to September, the average temperature, the average humidity, total rainfall, and total solar radiation were 26.2°C, 62%, 653.0 mm, and 1980.5 MJ/m^2^, respectively.

### Image acquisition

**Figure 11:**
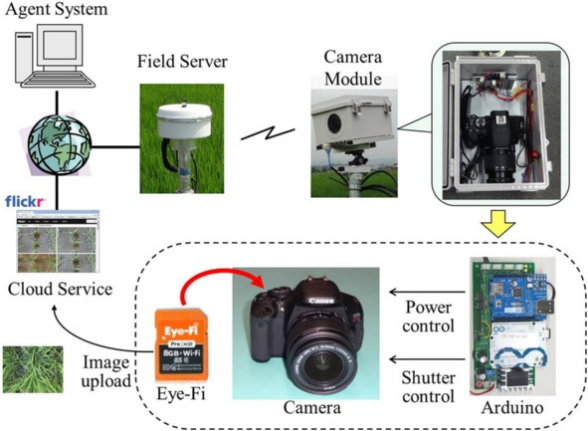
**The field server system used for image acquisition.**

A Field Server system [35,36] was used to acquire the experimental images (Figure 11). The camera module of the system is based on a digital single-lens reflex (DSLR) camera, the Canon EOS Kiss X5 camera, with an EF-S18-55 mm lens (Canon Inc., Tokyo) that provides high-quality and high-resolution (18 megapixels) image data. The power and shutter of the camera are controlled by a preprogrammed microcontroller board, the Arduino Uno (http://arduino.cc). The captured image data were sent to a free cloud service, Flickr (www.flickr.com) by a wireless uploading SD card, Eye-Fi (Eye-Fi, Inc., Mountain View) through WI-FI hotspots provided by the Field Servers at the field site. The Agent System [37] automatically grabs the images from the webpage of Flickr, arranges them, and saves them into a database at the National Agriculture and Food Research Organization using their EXIF data.

**Figure 12:**
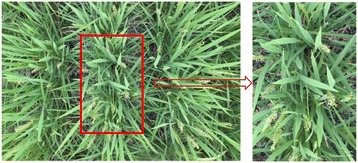
**Cropping of the original image.** The central region of each original image of the variety *Kinmaze* was cropped. The cropped region was corresponded to a field size of 30 × 45 cm that contained three rice plants.

The cameras are set to view the rice canopy from 2 m above the ground. At this distance, the image resolution is approximately 43 pixels/cm at the ground level and the resolution of crop images increases according to the crop growth. Using the system, time-series images of two paddy varieties were acquired every 5 min from 08:00 to 16:00 between days 84 and 91 after transplanting. Some of the images of the variety *Kinmaze* are missing because the system failed to acquire them. The failure was mainly due to the unstable network status in the field and was particularly obvious on day 86. Finally, a total of 645 images for *Kinmaze* (Dataset 1) and 768 images for *Kamenoo* (Dataset 3) were obtained. The images (5184 × 3456 pixels) corresponded to a field size of 138 cm × 98 cm and the number of the crops included in an image was around 30. Then, we cropped the original images of *Kinmaze* (Dataset 1) to the central regions in order to create a new time series image dataset named Dataset 2. The cropped image corresponded to a field size of 30 × 45 cm that contained three rice plants. Figure 12 shows the cropping, by which the original image of 5184 × 3456 pixels was cropped to a central region of 2001 × 1301 pixels. We used Dataset 2 to evaluate the influences of both the crop number included in an image and the distortion of the marginal area of the image caused by the camera lens on the accuracy of the flowering detection, comparing with the full size image dataset of *Kinmaze* (Dataset 1). To evaluate the flowering detection performance by the proposed method, the numbers of flowering panicles in all of the acquired images were counted visually.

### Flowering panicle detection

**Figure 13:**
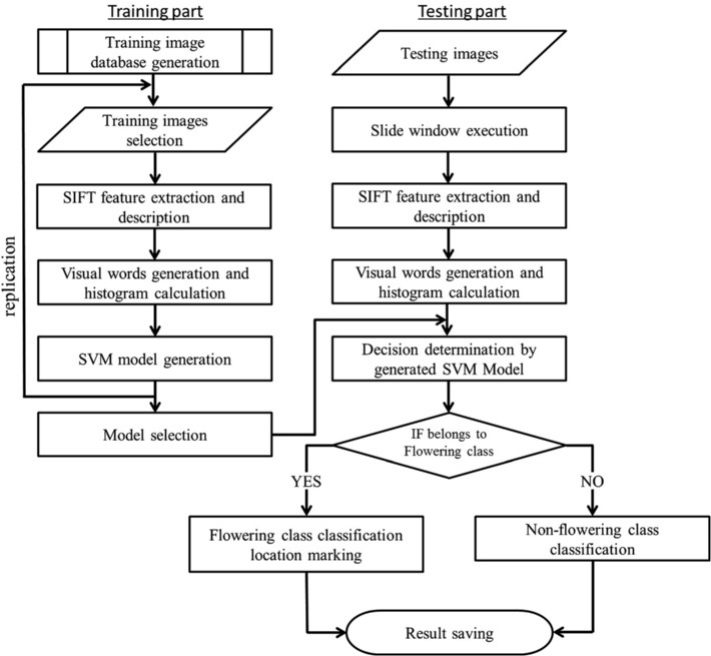
**Flowchart of the proposed flowering detection method.**

The full process is illustrated in Figure 13 and can be separated into two parts: training and testing. The process comprises the following steps:

1. Creating the training database by manually cropping the experimental images to yield rectangular regions. We created a database of training image patches of two classes, the positive class (flowering panicles) and the negative class (the background). Twenty one images from Dataset 1 were selected to obtain training data, considering the variation of the weather conditions in photographing (sunny, rainy, and cloudy conditions), the growth stage during the flowering period (initial, middle, and final flowering stages), and the positions (with and without occlusions and overlaps by other panicles and leaves). Finally, we obtained 300 image patches that contained part (s) of rice flowering panicles and 400 image patches that did not contain any part (s) of flowering panicles. An example of those training image patches are shown in Figure 14. Note that the sizes of the training image patches are not necessarily the same.

**Figure 14:**
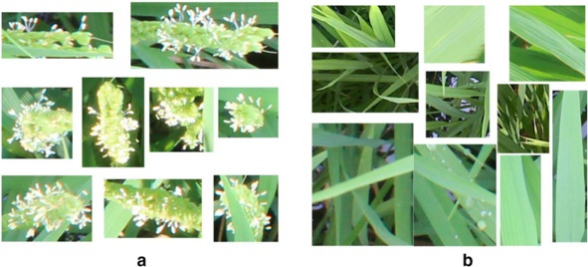
**Some examples of training image patches.** **(a)** Positive data which contain flowering parts of panicle (s); **(b)** Negative data which does not contain flowering parts of panicle (s), the training image patches were sampled from 21 images of Dataset 1.
2. Extracting local feature points and descriptors of those points from training image patches. In this study, we used SIFT descriptors [23] and dense sampling [38] to extract the points. In dense sampling, regular grid points with a space of M pixels are overlaid on an image and the SIFT descriptors are computed at each grid point of the image (Figure 15). In this study, we used M = 15 based on a preliminary test and used four circular support patches with radii r = 4, 6, 8, and 10 pixels to calculate scale-invariant SIFT descriptors. Consequently, each point was characterized by four SIFT descriptors, each of which comprised a 128-dimensional vector (Figure 15). The descriptor of each scale is based on a square with 16 patches [red squares in Figure 15 (b–e)]. The square is rotated to the dominant orientation of the feature point, and each patch in the square is described in the gradient magnitudes of eight different directions resulting in a total of 128 variables for each scale.

**Figure 15:**
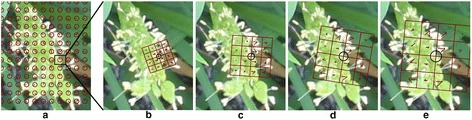
**An example of dense sampling and SIFT feature point description.** **(a)** SIFT descriptors are computed at regular grid points with a spacing of 15 pixels, as represented by the red circle; **(b** **-** **e)** At each point, SIFT descriptors are calculated on four different scales using four different radii: r = 4, 6, 8, and 10 pixels. The descriptor of each scale has 16 patches, represented by the red rectangles, which are rotated to the dominant orientation of the feature point. Each patch is described in gradient magnitudes of eight directions (red bins inside the red rectangles).
3. Generating visual words using the *k*-*means* method, which has been reported to perform well in object-recognition approaches [25,39]. The choice of the initial centroid position and the number of clusters (k) affects the resulting vocabulary in the *k*-*means* clustering method. In this study, we predefined *k* = 600 (number of visual words). We then ran *k*-*means* several times with random initial assignments of points as cluster centers, and used the best result to select the best-performing vocabulary. Note that these visual words do not contain location information of points.
4. Training the SVM as a flowering detection model, using the visual words as training data. SVM is one of the most popular machine learning models for object generic recognition. We used the SVM with a χ^2^ kernel, which is particularly powerful with data in histogram format [40,41]. A homogeneous kernel map was used to approximate the χ^2^ kernel to accelerate the learning process. The map transforms the data into a compact linear representation that reproduces the desired kernel to a very good level of approximation. This representation enables very fast linear SVM solvers [42]. The source code is available from the VLFeat open source library [43].
5. Verifying the performance of the generated SVM model for detecting the flowering parts of panicles in the test images. We used a sliding-window approach to apply the SVM model to the test images. The concept of the sliding window is to scan a whole test image without any overlaps using a predefined window size and then decide whether or not each scan window contains flowering parts, with reference to the trained model. In each scan window, the distribution of the visual words by the *k*-*means* method based on the entire set of sampling grid points where SIFT descriptors were calculated was used as an input to the generated SVM model. The most appropriate sliding-window size was determined by a preliminary test as 140 × 140 pixels for Dataset_1 and Dataset_2, and 170 × 170 pixels for Dataset_3, given that the size strongly affects flowering detection by the method.

The whole process was implemented using the software package MATLAB (MathWorks Inc., Natick) on a Microsoft Windows 8 PC with a 4-core CPU and 16 GB memory. Correlation analysis was performed with the statistical software package R (R Development Core Team, 2012).

### Training data selection

**Figure 16:**
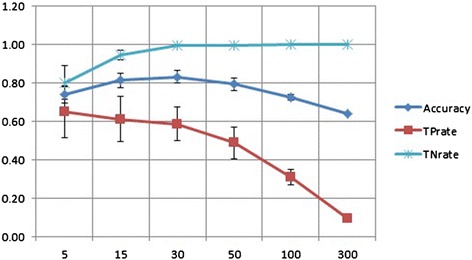
**Performance of SVM models under different numbers of training image patches.** Please see Table 1 for the definition of Accuracy, TPrate and TNrate. Considering Accuracy, TP rate and TN rate, the performance of the model is most well balanced when 30 training image patches were used.

Because the training image patches were manually selected, there was no guarantee that all of them provided “good” training data sets for training the flowering detection model. In addition, our preliminary test showed that the full use of the 300 positive and 400 negative training image patches did not provide the best performance compared with the use of the smaller number. Therefore, in lieu of using all the training image patches, we sought to determine how to select the most appropriate training image patches. We evaluated the accuracy of flowering detection using a different number of training image patches, for both positive and negative data with the aim of determining the optimal number, as follows: 5, 15, 30, 50, 100, and 300 (full use). Each set of images was randomly selected from the training image database with 10 replications, except when all 300 images were used. Then, using each of the training data sets, the SVM model was trained and its accuracy for flowering detection in the training image patches was evaluated. To evaluate the performance of the detection, three indices, accuracy, TP rate, and TN rate, were used. They are defined as follows:

$$ \mathrm{Accuracy}=\frac{TP + TN}{TP + FP + TN + FN} $$

$$ \mathrm{T}\mathrm{P}\ \mathrm{rate}=\frac{TP}{TP + FN} $$

$$ \mathrm{T}\mathrm{N}\ \mathrm{rate}=\frac{TN}{FP + TN} $$

where TP, TN, FP, and FN represent the numbers of true positives, true negatives, false positives, and false negatives of the confusion matrix, respectively. Accuracy measures the model detection ability for both flowering and background classes over the whole test data. The true positive rate, TP rate, measures the proportion of detected flowering images in the flowering class, whereas the true negative rate, TN rate, measures the detected background images in the background class. The means and standard deviations of the values from the 10 replications under different training image numbers are shown in Table 1 and Figure 16. The result shows that the performance of the model as measured by accuracy, TP rate, and TN rate is most well balanced with the training image number 30.

**Table 1:** **Relationship between the number of training images and the performance of flowering detection**

**Training number**	**5**	**15**	**30**	**50**	**100**	**300**
Accuracy^(+)^	0.74± 0.05	0.81± 0.04	0.83± 0.03	0.79± 0.03	0.73± 0.02	0.64
TP rate^(+)^	0.65± 0.13	0.61± 0.12	0.59± 0.09	0.49± 0.08	0.31± 0.04	0.09
TN rate^(+)^	0.8 ± 0.09	0.95± 0.03	0.99± 0.00	0.99± 0.00	1 ± 0.00	1.0

^+^Accuracy, TP rate, and TN rate, were defined as follows:

$$ \mathrm{Accuracy}=\frac{\mathrm{TP} + \mathrm{T}\mathrm{N}}{\mathrm{TP} + \mathrm{F}\mathrm{P} + \mathrm{T}\mathrm{N} + \mathrm{F}\mathrm{N}},\mathrm{T}\mathrm{P}\mathrm{rate}=\frac{\mathrm{TP}}{\mathrm{TP}+\mathrm{F}\mathrm{N}},\mathrm{T}\mathrm{N}\ \mathrm{rate}=\frac{\mathrm{TP}}{\mathrm{FP}+\mathrm{T}\mathrm{N}} $$

where TP, TN, FP, and FN represent the numbers of true positives, true negatives, false positives, and false negatives, respectively, of the confusion matrix.

**Figure 17:**
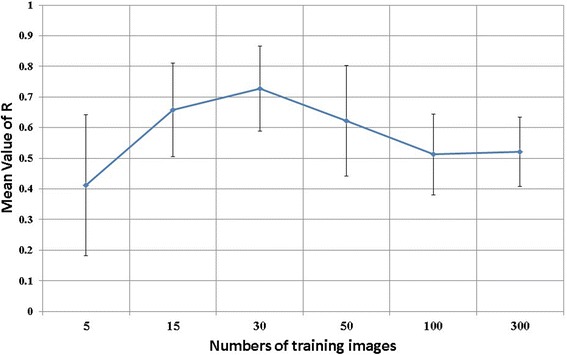
**Relationship between numbers of training image patches and flowering detection performance.** Performance is represented by the correlation coefficients between visually determined numbers of flowering panicles (FPN) and automatically detected numbers of flowering panicles (FCBN) in each case. The performance is best when 30 training image patches were used.

To verify the performance of flowering panicle detection by each model, we calculated the correlation coefficient (R) between visually determined flowering panicle numbers and numbers of blocks detected that contain flowering panicles (Figure 17). The R values increased with the number of training image patches until it reached 30, and then declined rapidly as the number increased. Thus, we again concluded that the training image number of 30 was optimal for flowering detection and used the training data set of 30 images that performed best among the 10 replicates in this study.

We originally expected that the full set of training image patches would perform best, but a much smaller number actually demonstrated the best performance in flowering detection. We can expect that the complexity of the background class generates widely varying SIFT descriptors within the class, and the more the training data, the more variation will appear. Such a variation in the SIFT features within a class may affect accuracy, although further studies are needed to identify the reason.

### Endnote

^a^http://park.itc.u-tokyo.ac.jp/nino-lab/labhome/PhenotypingTools/RiceFlower.html

## Additional file

Additional file 1:
**A demo video that shows flowering panicle detection on Dataset_1.**

## Abbreviation

SIFT: Scale-Invariant feature transform
BoVWs: Bag of visual words
SVM: Support Vector Machine
DSLR: Digital Single-Lens Reflex
TP: True positive
TN: True negative
TPrate: True positive rate
TNrate: True negative rate
FBN: The number of the blocks which are judged to contain the flowering parts of panicles
FCBN: The number of the regions of connected blocks
FPN: The number of visually counted flowering panicles
